# Delays and declines in seasonal influenza vaccinations due to Hurricane Harvey narrow annual gaps in vaccination by race, income and rurality

**DOI:** 10.1017/ice.2022.27

**Published:** 2022-12

**Authors:** Margaret A. Carrel, Gosia S. Clore, Seungwon Kim, Michihiko Goto, Eli N. Perencevich, Mary Vaughan Sarrazin

**Affiliations:** 1 Department of Geographical & Sustainability Sciences, University of Iowa, Iowa City, Iowa; 2 Department of Internal Medicine, University of Iowa, Iowa City, Iowa; 3 Center for Access & Delivery Research and Evaluation (CADRE), Iowa City Veterans’ Affairs Health Care System, Iowa City, Iowa

## Abstract

**Objective:**

Temporal overlap of the Atlantic hurricane season and seasonal influenza vaccine rollout has the potential to result in delays or disruptions of vaccination campaigns. We documented seasonal influenza vaccination behavior over a 5-year period and explored associations between flooding following Hurricane Harvey and timing and uptake of vaccines, as well as how the impacts of Hurricane Harvey on vaccination vary by race, wealth, and rurality.

**Design:**

Retrospective cohort analysis.

**Setting:**

Texas counties affected by Hurricane Harvey.

**Patients:**

Active users of the Veterans’ Health Administration in 2017.

**Methods:**

We used geocoded residential address data to assess flood exposure status following Hurricane Harvey. Days to receipt of seasonal influenza vaccines were calculated for each year from 2014 to 2019. Proportional hazards models were used to determine how likelihood of vaccination varied according to flood status as well as the race, wealth, and rural–urban residence of patients.

**Results:**

The year of Hurricane Harvey was associated with a median delay of 2 weeks to vaccination and lower overall vaccination than in prior years. Residential status in flooded areas was associated with lower hazards of influenza vaccination in all years. White patients had higher proportional hazards of influenza vaccination than non-White patients, though this attenuated to 6.39% (hazard ratio [HR], 1.0639; 95% confidence interval [CI], 1.034–1.095) in the hurricane. year.

**Conclusions:**

Receipt of seasonal influenza vaccination following regional exposure to the effects of Hurricane Harvey was delayed among US veterans. White, non–low-income, and rural patients had higher likelihood of vaccination in all years of the study, but these gaps narrowed during the hurricane year.

The Atlantic hurricane season, extending from June 1 to November 30, overlaps with the annual rollout of seasonal influenza vaccines in the United States; the Centers for Disease Control & Prevention (CDC) recommends influenza vaccination occur by the end of October. As the frequency and intensity of hurricanes increases in coming decades due to global climate change, such storm events could negatively impact influenza vaccination rates just as they do other forms of healthcare utilization and health outcomes.^1–8^ Individuals exposed to hurricanes, flooding or other natural hazards may be displaced from their typical care providers, have little time to focus on preventative care behaviors, or experience greater risk of influenza exposure through sheltering with large groups of people.^9^ Following Hurricane Katrina, for instance, the CDC recommended that displaced persons living in crowded group settings receive an influenza vaccine.^10^

Seasonal influenza vaccine uptake in the United States varies according to social determinants and geographically. Studies in several health settings have shown higher influenza vaccination rates among White patients versus non-White patients, findings linked to structural racism in healthcare settings, social norms surrounding vaccine behavior and perceptions of both risk and trust.^11–17^ Wealth is positively correlated with influenza vaccination in the United States, with higher-income patients having higher vaccination rates.^11,15,18,19^ Rural residence has also been associated with higher influenza vaccination in US adults.^11,20^ The greatest predictor of influenza vaccination among US adults in the Behavioral Risk Factors Surveillance System (BRFSS) was healthcare coverage.^21^

The Veterans’ Health Administration (VHA) is the largest integrated healthcare system in the United States. In addition to providing care at medical centers and community-based outpatient clinics, the VHA partners with tens of thousands of community care providers, such as urgent clinics and retail pharmacies. Through the community care network, Veterans can receive free influenza vaccinations at non-VHA locations. VHA data from 2011 to 2021 indicated annual variation in influenza vaccination rates, ranging from 27% to 34% of VHA users in each year getting vaccinated.^22,23^ Moreover, >98% of veteran vaccinations took place in outpatient settings. The veteran population in the United States is older and has higher rates of comorbidities than the general population; thus, many veterans would fall into the Healthy People 2020 categories for whom influenza vaccinations would provide the greatest benefit.^24,25^

Hurricane Harvey made landfall in late August 2017, and it caused catastrophic flooding across east Texas. Veterans in the 41 Texas counties declared eligible for individual assistance by the Federal Emergency Management Agency (FEMA) are served by 1 VHA medical center, 7 community-based outpatient clinics and hundreds of pharmacies and urgent care centers in the community care network. Utilizing veteran health records, we examined how Hurricane Harvey affected receipt of influenza vaccination. We assessed delays in vaccination and explored how this varies by residential flood status and rurality as well as by the race and income of patients. Given that vaccination can occur at many locations but cannot be replaced by telehealth provision of care, and that influenza vaccination has direct and indirect health benefits, understanding how hurricane exposure, coupled with social and geographic determinants of vaccination uptake, influences seasonal influenza vaccination is key to informing public health disaster responses.

## Methods

A cohort of veterans who were active users of the VHA for primary care and resided in the 41-county hurricane-affected study area in 2017 was generated from the VA Corporate Data Warehouse (CDW). All veteran residential addresses are geocoded to a latitude/longitude; patients were excluded from the cohort if the VA geocoded them to a post office box, ZIP code, or town rather than a specific street address or if their address matched the location of a state-licensed nursing home or other group facility. Electronic health records for the veteran cohort were obtained for July 1, 2014, through June 30, 2019. Assessment of influenza vaccination was made for 5 separate years, spanning July 1 through June 30 of the following calendar year (ie, a “flu year”), to account for seasonality in influenza vaccine availability and typical influenza seasons. Influenza vaccinations were captured using current procedural terminology (CPT) codes; no distinction was made between high-dose, quadrivalent, or other types of vaccine formulations.

In addition to receipt of influenza vaccination in each of 5 flu years, the CDW was queried for data on veteran sex, age, race, comorbidities, income, and rurality. Also, 14 comorbidities were identified to create a Charlson score for each veteran.^26^ VHA enrollment priority was used to categorize patients as having service-connected disability >50%, service-connected disability <50%, low income, and other for this study. Veterans are categorized as low income (priority category 5) if they do not have a service-related disability and have income below a VA determined adjusted income limit (based on resident ZIP code) or are eligible for Medicaid. All enrollment priority categories other than low income were collapsed for the study. Rural, highly rural, or urban designations are assigned by the VHA based upon Rural–Urban Commuting Area (RUCA) codes for census tracts where veterans live. Rural and highly rural categories were collapsed for the study.

Residential flood status of veterans during Hurricane Harvey was assessed using a flood depth grid developed by FEMA (Supplementary Fig. 1 online).^27^ The depth grid has 3-m by 3-m horizontal resolution and is available as a geographic information systems (GIS) raster data set. The latitude and longitude of veteran residence assigned by VHA was overlaid onto the flood raster using ArcMap 10.7.1 (Esri, Redlands, CA). Veterans were designated as residentially flooded or not flooded based upon their relationship to the flood grid.

**Fig. 1.**
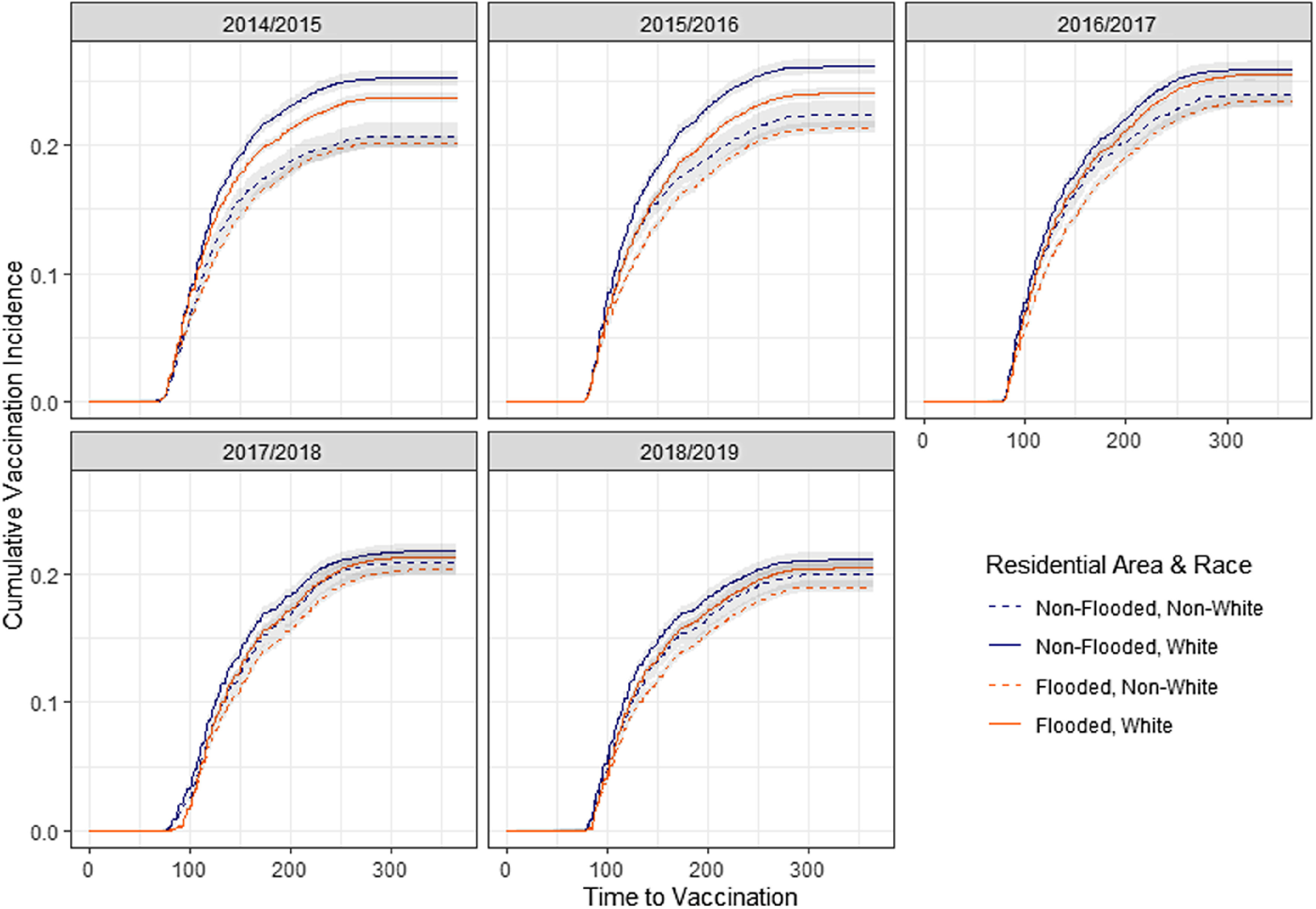
Cumulative vaccinations over time for veterans in 5 flu years, stratified by flood status during Hurricane Harvey and by race.

Differences in sociodemographic characteristics between veterans who received an influenza vaccination during the study period and those who did not were assessed via χ^2^ tests. The percentages of veterans vaccinated in each flu year were calculated, as were the means, medians and ranges of days to vaccination in each flu year to evaluate potential delays in vaccine uptake. The numbers and percentages of veterans receiving 0–5 vaccinations during the study period were charted to assess the frequency of veterans having the recommended annual receipt of an influenza vaccine.

Survival curves measuring probability of receiving vaccination over time in each flu year were generated, stratified by flood status, race, priority category 5 status and rurality, and inverted to generate cumulative incidence estimates and confidence intervals of vaccination over time in each of the 5 flu years. Patients were censored if they died during the study period following Hurricane Harvey. Survival analysis was conducted in SAS EG version 8.2 software (SAS Institute, Cary, NC) and results were charted in R (CRAN, Vienna, Austria).

Cox’s proportional hazards models were used to assess the likelihood and timing of influenza vaccinations in the year of Hurricane Harvey versus other years, stratified by flood status, race, income, and rurality. Hazard of vaccination was assessed at the monthly timescale to examine the potential for differential impacts of the hurricane over time. No confounders were added to the model as all veterans, regardless of age or gender or comorbidities, are encouraged to receive the influenza vaccine. We present hazard ratios (HRs) with 95% confidence intervals (CIs) for Cox proportional hazards regression analyses and use the phrases likelihood and probability to avoid negative connotations being associated with vaccination. All hazards modeling was performed using SAS EG version 8.2 software. The study was approved by the Institutional Review Board at the University of Iowa.

## Results

### Overall vaccination pattern

There were 102,407 active users of VHA primary care residing in the 41 Texas counties most impacted by Hurricane Harvey. Of those, 2,549 (2.48%) of 102,407 were not included in subsequent analysis because of poor residential geocoding or residence in a group facility. Among the veteran cohort exposed to Hurricane Harvey, 47,421 (47.49%) of 99,858 never received an influenza vaccination during the 5-year study period (Table 1). Additionally, repeated receipt of influenza vaccinations was low (Supplementary Fig. 2 online). Also, 20.54% of the cohort received only a single influenza vaccine, and only 2% received an influenza vaccination during all 5 years of the study.

**Table 1.** Characteristics of Veteran Patients Included in the Cohort

Variable	Total	%	Total Ever Vaccinated	% Ever Vaccinated	*P* Value
Total cohort	99,858	100	52,437	52.51	
**Sex**					
Female	9,927	9.94	4,861	48.97	<.0001
Male	89,931	90.06	47,576	52.9	<.0001
**Age**					
Mean (SD)	58	(±16.8)	61	±15.5	<.0001
<50 y	30,469	30.51	12,458	40.89	<.0001
50–59 y	14,268	14.29	7,405	51.9	0.114
60–69 y	26,598	26.64	16,270	61.17	<.0001
70–79 y	19,977	20.01	12,050	60.32	<.0001
>80 y	8,546	8.56	4,254	49.78	<.0001
**Race**					
White	63598	63.69	34,211	53.79	<.0001
Black	28952	28.99	14,767	51.01	<.0001
Multiple	1179	1.18	599	50.81	0.238
Other	2066	2.07	1,107	53.58	0.325
Missing	4063	4.07	1,753	43.15	<.0001
**Charlson score**					
0	22,024	22.06	8,336	37.85	<.0001
1	10,905	10.92	5,085	46.63	<.0001
2	11,368	11.38	5,861	51.56	0.03
3	12,398	12.42	6,707	54.1	2E-04
4	11,225	11.24	6,364	56.69	<.0001
5	8,599	8.61	5,174	60.17	<.0001
≥6	23,339	23.37	14,910	63.88	<.0001
**Hurricane Harvey flood status**					
Flooded	73,120	73.22	38,231	52.29	0.018
Not flooded	26,738	26.78	14,206	53.13	0.018
**Rurality**					
Urban	74,988	75.09	38,403	51.21	<.0001
Rural	24,870	24.91	14,034	56.43	<.0001
**Priority category**					
1 (disability > 50%, Medal of Honor)	38,851	38.91	21,511	55.37	<.0001
2–4 (disability <50%, POW, Purple Heart, catastrophically disabled)	23,621	23.65	11,872	50.26	<.0001
5 (low income)	19,748	19.78	10,399	52.66	0.644
6–8 (other)	17,638	17.66	8,655	49.07	<.0001

Note. POW, prisoner of war.

**Fig. 2.**
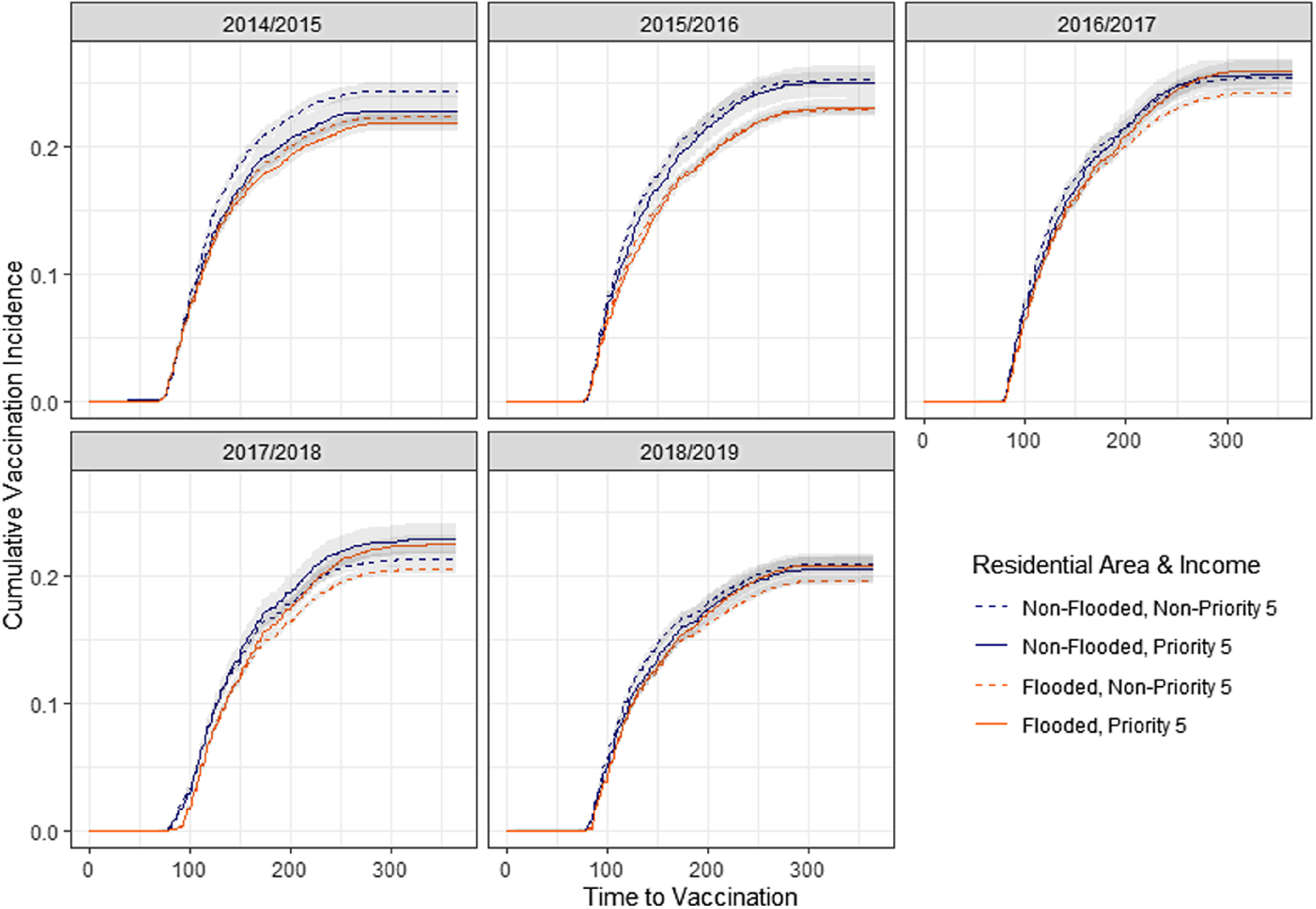
Cumulative vaccinations over time for veterans in 5 flu years, stratified by flood status during Hurricane Harvey and by priority status (income proxy).

Men made up a larger percentage of the vaccinated cohort than the unvaccinated cohort, as did older veterans and White veterans. Vaccinated patients were more likely to have a high Charlson comorbidity score and rural residence. Veterans residing in areas not flooded during Hurricane Harvey had slightly higher rates of vaccination. No significant differences in the low-income priority category were observed (Table 1).

### Impact of Hurricane Harvey on vaccinations

Overall receipt of influenza vaccinations in the study cohort was low. During the 5 years of the study, the percentage of veterans who were vaccinated never exceeded 25% (Table 2). The percent of veterans receiving their influenza vaccine was lower during the year of Hurricane Harvey than the preceding 3 years (Table 2). In non-Hurricane Harvey years, influenza vaccinations occurred across nearly the entire span of the vaccination year, from July through June of the following year, with the bulk of vaccinations taking place in the fall months. The year Hurricane Harvey made landfall, 2017–2018, the mean time to vaccination increased by >1 week compared to other years, and the median time increased by close to 2 weeks.

**Table 2.** Total Patients in the Study Cohort Receiving Influenza Vaccines in Each Year and Days to Vaccination Across the Study Years

Year	Total (%)	Mean	SD	Median	Minimum	Maximum	IQR
2014/2015	22,723 (22.75)	128.77	45.74	115	0	359	93–153
2015/2016	23,534 (23.57)	138.30	51.26	121	16	358	97–167
2016/2017	24,809 (24.84)	141.51	53.46	124	14	348	98–172
2017/2018	20,864 (21.46)	150.66	49.71	136	39	354	111–181
2018/2019	19,334 (20.48)	141.56	51.62	123	2	352	101–169

Note. SD, standard deviation; IQR, interquartile range.

During the year of Hurricane Harvey, the pattern of influenza vaccinations is similar among veterans with residential flood exposure compared to those without. In contrast, in non-hurricane years the rates of vaccination are lower in veterans with residential flood exposure (Supplementary Fig. 3 online). In the year of Hurricane Harvey, the peak rate for flu shots is about the same for flooded and nonflooded veterans (∼1,900 of 100,000) and happens at about the same time (∼week 8 after Harvey), but the uptick in influenza vaccines happens sooner in the nonflooded cohort.

**Fig. 3.**
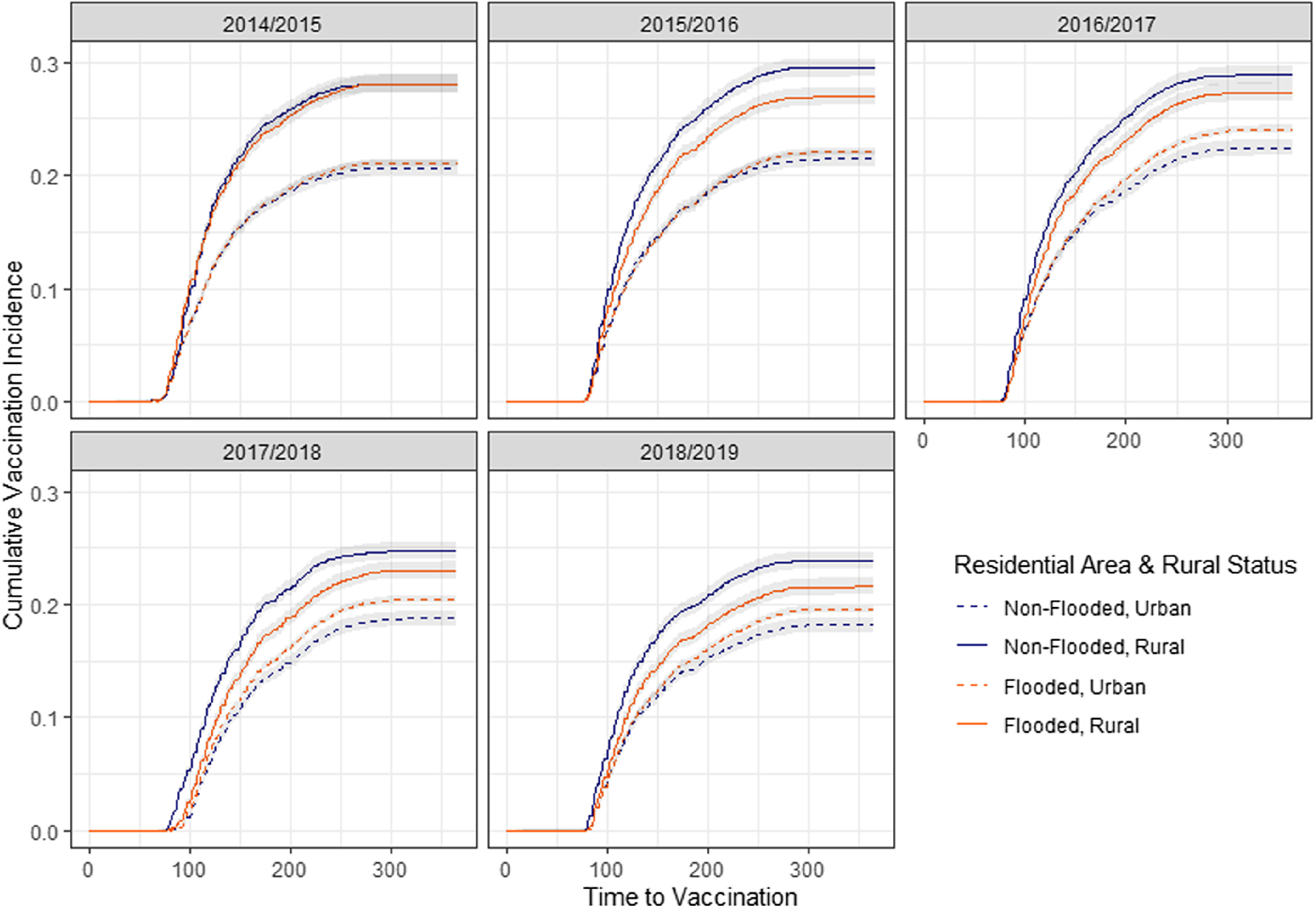
Cumulative vaccinations over time for veterans in 5 flu years, stratified by flood status during Hurricane Harvey and by rurality.

### Stratified analysis by race, priority category, and rurality

In all years of the study, White veterans had higher vaccination rates over time than did non-White veterans (Fig. 1). White veterans living in areas that did not flood during Hurricane Harvey had higher vaccination rates than White veterans living in flooded areas. The lowest vaccination rates across all years were observed in non-White veterans whose residences flooded during Hurricane Harvey.

Cumulative vaccination over time is similar across low-income, priority 5 category veterans and those in other priority categories (Fig. 2). During the year Hurricane Harvey made landfall (2017–2018), influenza vaccination among priority 5 veterans who experienced residential flooding began later than their nonflooded counterparts. At the end of the 2017–2018 flu year, vaccination incidence was higher among priority 5 category veterans, regardless of flood status, than non–priority 5 category veterans.

Influenza vaccinations among rural veterans, regardless of flood status, have higher cumulative vaccination incidence than do urban veterans in all years of the study (Fig. 3). These differences are not as substantial during the year of Hurricane Harvey. Rural veterans who lived in flooded areas had lower vaccination incidence over time than did nonflooded rural veterans. In contrast, vaccination incidence was lower in all years among nonflooded, urban veterans than their flooded, urban counterparts.

Irrespective of flood status, in all years of the study, White veterans had higher probability of receiving an influenza vaccine than did non-White veterans (Table 3). This probability was greater at 15.33% (hazard ratio [HR], 1.1533; 95% confidence interval [CI], 1.138–1.169) in non-Hurricane Harvey years than in 2017–2018, when White veterans were 6.39% (HR, 1.0639; 95% CI, 1.034–1.095) more likely to receive a vaccine. The differences between White and non-White vaccinations significantly diminished (HR, 0.9225; 95% CI, 0.894–0.952) in Hurricane Harvey year versus other years.

**Table 3.** Absolute and Relative Hazard ratios for Influenza Vaccination for Veterans Stratified by Race, Income, and Rurality

Variable	Hurricane Harvey Year	Non-Hurricane Harvey Year	Relative Change
White: Non-White	1.06 (1.03–1.10)	1.15 (1.14–1.17)	0.92 (0.89–0.95)
Priority 5: Non–priority 5	0.97 (0.90–1.04)	0.97 (0.93–1.00)	1.08 (1.04–1.12)
Rural: Urban	1.23 (1.19–1.27)	1.30 (1.28–1.32)	0.94 (0.91–0.98)
Flooded: Nonflooded	0.95 (0.93–0.98)	0.92 (0.91–0.93)	1.04 (1.00–1.07)

Low-income veterans in priority category 5 had 3.25% (HR, 0.9675; 95% CI, 0.904–1.036) lower influenza vaccination likelihood in the year of Hurricane Harvey than veterans in other priority categories, though this was not significant. Significantly lower influenza vaccination probability among low-income veterans was observed in other years of the study (HR, 0.97; 95% CI, 0.93–0.99). Differences between low-income and other veterans increased during the year of Hurricane Harvey compared to other years (HR, 1.0769; 95% CI, 1.038–1.118).

In all years of the study, veterans residing in rural areas, regardless of flood exposure, had higher influenza vaccination likelihood than urban veterans: 22.89% higher in the Hurricane Harvey flu year (HR, 1.23; 95% CI, 1.19–1.23) and 30.17% higher in other years (HR, 1.32; 95% CI, 1.28–1.32). Hurricane Harvey was associated with significantly decreased differences in influenza vaccination probability between rural and urban veterans (HR, 0.94; 95% CI, 0.91–0.98).

Veterans residing in flooded areas had lower influenza vaccination likelihood than their nonflooded counterparts in all years of the study. In non-Harvey years, veterans residing in flooded areas had 7.86% (HR, 0.92; 95% CI, 0.91–0.93) lower vaccination likelihood than nonflooded veterans. The difference in vaccination between flooded and nonflooded veterans was smaller, 4.59% (HR, 0.95; 95% CI, 0.93–0.98) during the 2017–2018 flu year. This change between Harvey/non-Harvey years in flooded versus nonflooded areas was significant (HR, 1.04; 95% CI, 1.00–1.07).

### Impact of residential flood status

Overall, the Hurricane Harvey flu year was associated with reductions in influenza vaccines for all veterans in the study area. However, the relative decrease in influenza vaccination probability in Hurricane Harvey years relative to other years was larger among veterans residing in nonflooded areas (HR, 0.87; 95% CI, 0.85–0.90) compared to the decrease among veterans living in flooded areas (HR, 0.90; 95% CI, 0.89–0.92) (Table 4).

**Table 4.** Absolute and Relative Hazard Ratios for Influenza Vaccination Among Veterans in Flooded Versus Nonflooded Areas in Hurricane Harvey Flu Year (2017–2018) and for the Impact of Residential Flooding in Hurricane Harvey Flu Year Versus All Other Years, Stratified by Race, Income, and Rurality

Variable	Hurricane Harvey Year Impact, Relative Hazard Ratio (95% CI)		Relative Change
	Flooded Area	Nonflooded Area	
Overall	0.90 (0.89–0.92)	0.87 (0.85–0.89)	1.04(1.00–1.07)
White	0.87 (0.86–0.89)	0.85 (0.83–0.88)	1.03 (0.98–1.07)
Non-White	0.94 (0.92–0.97)	0.93 (0.88–0.99)	1.01 (0.94–1.08)
Priority 5	0.95 (0.91–0.98)	0.95 (0.89–1.01)	1.00 (0.93–1.08)
Non–Priority 5	0.89 (0.87–0.91)	0.85 (0.83–0.88)	1.04 (1.00–1.08)
Rural	0.84 (0.81–0.88)	0.87 (0.83–0.90)	0.97 (0.92–1.03)
Urban	0.91 (0.89–0.93)	0.87 (0.84–0.91)	1.05 (0.99–1.10)

Note. CI, confidence interval.

White veterans experienced slightly smaller decreases in vaccination likelihood in flooded areas (HR, 0.87; 95% CI, 0.86–0.90) compared to nonflooded areas (HR, 0.85; 95% CI, 0.83–0.88) during the year of Hurricane Harvey. This pattern was similar for non-White patients (HR, 0.87; 95% CI, 0.86–0.90 in flooded areas; HR, 0.85; 95% CI, 0.83–0.88 in non-flooded areas). Low-income, priority category 5 veterans in both flooded and nonflooded areas saw lower influenza vaccination probability during the Hurricane Harvey year, though this was not significant for nonflooded veterans. Among nonpriority category 5 veterans, the year of Hurricane Harvey was associated with greater declines in influenza vaccination probability among those not living in flooded areas than those in flooded areas.

Residential flood exposure during Hurricane Harvey had opposite impacts in rural versus urban veterans. Among rural veterans, larger declines during Hurricane Harvey year were observed in influenza vaccination likelihood for nonflooded areas (HR, 0.87; 95% CI, 0.83–0.90) than flooded areas (HR, 0.84; 95% CI, 0.81–0.88). In contrast, among urban veterans, the declines in influenza vaccination likelihood were larger in flooded areas (HR, 0.91; 95% CI, 0.90–0.93) than nonflooded areas (HR, 0.87; 95% CI, 0.84–0.91).

### Varying impacts over time

Across all strata, the likelihood of vaccination was lower in the 2 months following Hurricane Harvey compared to those same months in other years (Supplementary Table 1 online).

## Discussion

Influenza vaccination rates from 2014–2019 among Texas veterans residing in areas impacted by Hurricane Harvey were low: nearly half never received a vaccine and only 2% received a vaccination in all 5 years of the study. Annual vaccination percentages ranged from 20.48% to 24.84%, with the lowest rates observed in the year of Hurricane Harvey and the subsequent year. This is lower than nationwide VHA influenza vaccination rates in the same years.^22,23^ It is also lower than estimates of influenza vaccination during the study period in the US adult population overall, although Texas does have lower vaccination rates than most other states.^28^ This year of lower vaccination coincides with a year of high influenza hospitalizations within the VHA and high percentages of positive influenza tests.^23^

No receipt of an influenza vaccination in the study period was observed more frequently in females, younger veterans, non-White veterans, urban veterans, and those with lower Charlson comorbidity scores. No significant differences between ever-vaccinated and never-vaccinated veterans were observed among those in low-income categories versus those in other categories. Never receiving an influenza vaccination was more common among veterans in areas that flooded during Hurricane Harvey.

Longer times to vaccination were observed during the year of Hurricane Harvey. Mean time to vaccination increased by over a week and median time by over 2 weeks. Likelihood of vaccination remained lower in the 2 months following Hurricane Harvey than during that same period in other years. Later receipt of influenza vaccinations has been associated with reduced efficacy.^29^

The year of Hurricane Harvey was associated with declines in influenza vaccination across all strata: race, income, rurality, and flood exposure. The largest declines were observed among White veterans, veterans not in the low-income category, and rural veterans. However, in all years of the study, these groups had higher influenza vaccination than non-White, low-income, and urban veterans. The impacts of Hurricane Harvey, then, were to decrease inequity in receipt of influenza vaccinations across the veteran patient population.

This study had several limitations. In understanding the impact of flooding on vaccination, there were no clear measures of the duration of the flood events or how they varied across the study area. The inclusion of flood as a dichotomous variable potentially obscured the fact that some veterans were dealing with the impacts of flooding for longer than others and that this could have affected the timing or overall receipt of vaccination. Another limitation of the study is that low-income veterans with high levels of disability are not included in priority category 5, so we may be underestimating differences between low-income and non–low-income veterans. Additionally, we dichotomize race into White and non-White, which could mask differences in vaccination among non-White race and ethnicity groups. No multivariable analysis was conducted, so potential confounding effects or interactions are unknown. Finally, we only consider vaccination behavior among veterans who were active users of the VHA for primary care; thus, we may be underestimating vaccination behaviors among veterans who receive primary care outside the VHA system.

Hurricane Harvey was associated with delays and declines in the receipt of influenza vaccination among veterans. Although vaccinations declined across all groups, the impact of the hurricane was not to exacerbate existing differences in vaccination rates across race, income, and rurality but rather to decrease those differences. Although it is desirable for inequity in preventive care to decrease, achieving this by lowering care provision across all groups is not preferable. Although locations providing influenza vaccinations to veterans are widely distributed, perhaps the use of mobile clinics during nondisaster periods could decrease the pervasively observed differences in vaccination by race, income and rurality.^30,31^

## Supplementary material

For supplementary material accompanying this paper visit http://doi.org/10.1017/ice.2022.27. click here to view supplementary material
