# Supplementary material for: Delays and declines in seasonal influenza vaccinations due to Hurricane Harvey narrow annual gaps in vaccination by race, income and rurality
Source: Infect Control Hosp Epidemiol. 2022 Mar 16;43(12):1833–9. doi: 10.1017/ice.2022.27 (PMC9753087; doi:10.1017/ice.2022.27)
Supplement: Supplementary file 1 [file S0899823X22000277sup001.docx]

**Supplementary Material**

**
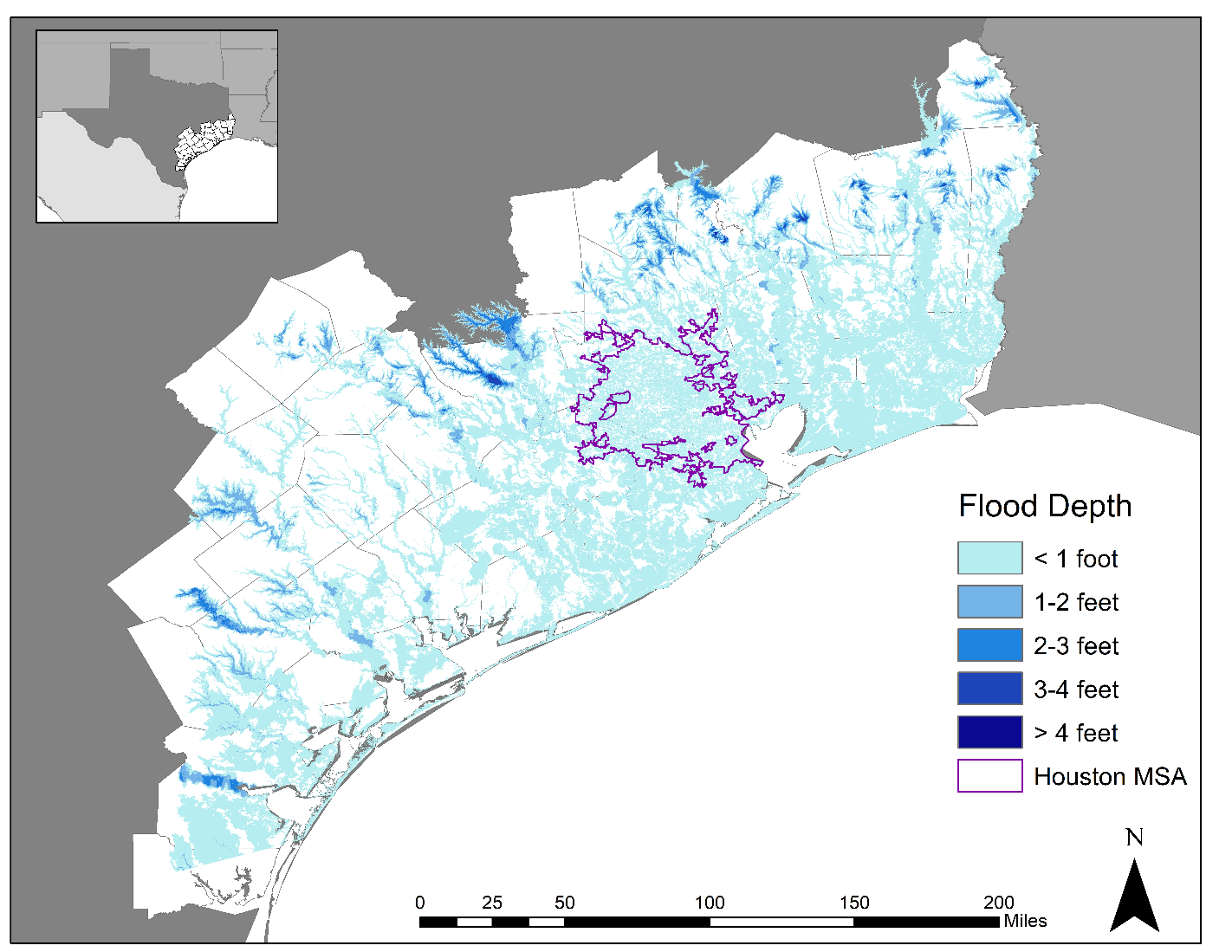
**

**Supplementary Figure 1: Study area, Texas counties eligible for individual assistance via FEMA following Hurricane Harvey, and the extent of flooding associated with Hurricane Harvey. The Houston metropolitan statistical area is shown for reference.**

**
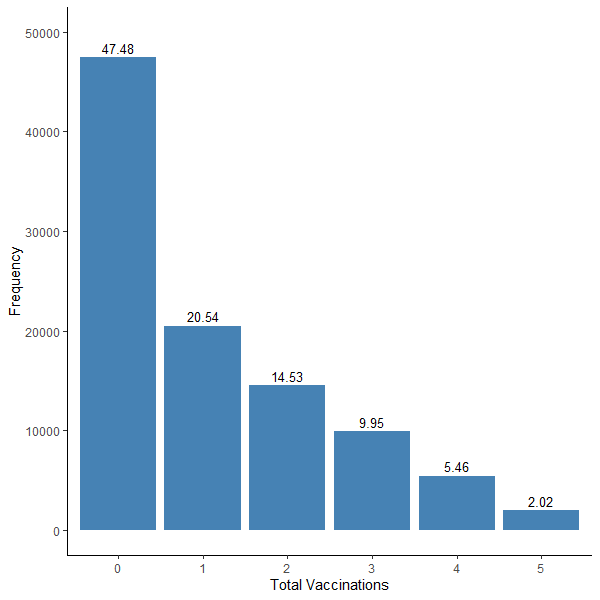
**

**Supplementary Figure 2: Vaccine receipt by individual veteran patients across the study period (2014-2019). Percent of the total cohort is shown above the frequency bar.**

**
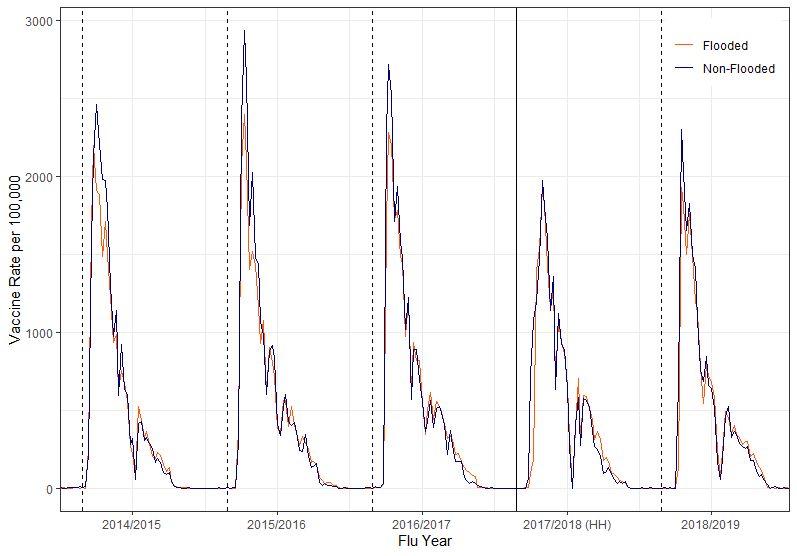
**

**Supplementary Figure 3: Influenza vaccination over time, stratified by flood status. The week Hurricane Harvey made landfall is indicated with a solid line, and that same week in prior and subsequent years is indicated by a dashed line.**

**Supplementary Table 1. Absolute and relative monthly hazard ratios for influenza vaccination among veterans in Hurricane Harvey flu year (2017/2018) versus other years, stratified by flood status, race, income and rurality.**

| Month (Harvey Year vs Non-Harvey Year) | Flood | Non-Flood | Relative | White | Non-White | Relative |
| --- | --- | --- | --- | --- | --- | --- |
| July | 0.009 (0.007,0.012) | 0.021 (0.014, 0.032) | 0.436 (0.269, 0.707) | 0.013 (0.010,0.017) | 0.009 (0.006, 0.013) | 1.438 (0.916, 2.260) |
| August | 0.218 (0.201,0.236) | 0.281 (0.247, 0.320) | 0.775 (0.665, 0.903) | 0.241 (0.221,0.262) | 0.223 (0.198, 0.250) | 1.081 (0.935, 1.249) |
| September | 0.416 (0.396,0.436) | 0.477 (0.443, 0.514) | 0.871 (0.798, 0.951) | 0.435 (0.414,0.457) | 0.427 (0.398, 0.457) | 1.020 (0.937, 1.110) |
| October | 0.607 (0.589,0.625) | 0.650 (0.622, 0.680) | 0.933 (0.884, 0.984) | 0.616 (0.597,0.634) | 0.625 (0.598, 0.652) | 0.985 (0.935, 1.038) |
| November | 0.976 (0.959,0.994) | 0.960 (0.931, 0.989) | **1.017 (0.982, 1.054)** | 0.953 (0.934,0.971) | **1.009 (0.983, 1.036)** | 0.944 (0.914, 0.975) |
| December | **1.157 (1.133,1.183)** | **1.103 (1.063, 1.145)** | 1.049 (1.005, 1.096) | **1.113 (1.088,1.140)** | 1.198 (1.162, 1.235) | 0.930 (0.895, 0.966) |
| January | 1.336 (1.301,1.372) | 1.241 (1.184, 1.300) | 1.077 (1.020, 1.136) | 1.270 (1.233,1.308) | 1.384 (1.333, 1.437) | 0.918 (0.875, 0.963) |
| February | 1.513 (1.465,1.563) | 1.374 (1.299, 1.453) | 1.102 (1.033, 1.175) | 1.424 (1.375,1.475) | 1.569 (1.499, 1.642) | 0.907 (0.857, 0.961) |
| March | 1.688 (1.626,1.752) | 1.503 (1.408, 1.603) | 1.124 (1.043, 1.211) | 1.574 (1.511,1.640) | 1.752 (1.662, 1.847) | 0.899 (0.841, 0.960) |
| April | 1.864 (1.787,1.944) | 1.629 (1.515, 1.752) | 1.144 (1.052, 1.244) | 1.724 (1.646,1.805) | 1.936 (1.823, 2.055) | 0.891 (0.826, 0.960) |
| May | 2.036 (1.943,2.133) | 1.752 (1.616, 1.898) | 1.162 (1.060, 1.275) | 1.870 (1.777,1.967) | 2.116 (1.981, 2.261) | 0.884 (0.813, 0.960) |
| June | 2.208 (2.099,2.323) | 1.872 (1.716, 2.042) | 1.180 (1.067, 1.305) | 2.014 (1.906,2.129) | 2.297 (2.137, 2.468) | 0.877 (0.801, 0.960) |
|  |  |  |  |  |  |  |
| Month (Harvey Year vs Non-Harvey Year) | P5 | Non-P5 | Relative | Rural | Urban | Relative |
| July | 0.010 (0.006,0.016) | 0.012 (0.009, 0.015) | 0.830 (0.489, 1.409) | 0.030 (0.020,0.045) | 0.008 (0.006, 0.010) | 3.717 (2.299, 6.012) |
| August | 0.230 (0.198,0.268) | 0.235 (0.217, 0.254) | 0.980 (0.826, 1.161) | 0.310 (0.273,0.353) | 0.209 (0.193, 0.227) | 1.482 (1.273, 1.724) |
| September | 0.437 (0.400,0.477) | 0.431 (0.412, 0.451) | 1.013 (0.918, 1.119) | 0.500 (0.465,0.538) | 0.407 (0.388, 0.427) | 1.228 (1.126, 1.340) |
| October | 0.636 (0.601,0.672) | 0.615 (0.598, 0.632) | 1.034 (0.972, 1.099) | 0.662 (0.634,0.691) | 0.601 (0.584, 0.619) | 1.101 (1.044, 1.160) |
| November | **1.019 (0.986,1.054)** | 0.962 (0.945, 0.979) | 1.060 (1.021, 1.101) | 0.941 (0.913,0.970) | 0.982 (0.964, 0.999) | 0.959 (0.926, 0.993) |
| December | 1.206 (1.159,1.255) | **1.128 (1.105, 1.152)** | 1.069 (1.022, 1.118) | **1.067 (1.028,1.108)** | **1.169 (1.144, 1.195)** | 0.913 (0.874, 0.953) |
| January | 1.391 (1.324,1.461) | 1.291 (1.257, 1.326) | 1.077 (1.019, 1.139) | 1.186 (1.132,1.244) | 1.356 (1.320, 1.392) | 0.875 (0.829, 0.924) |
| February | 1.574 (1.483,1.670) | 1.451 (1.406, 1.498) | 1.084 (1.014, 1.160) | 1.301 (1.230,1.376) | 1.541 (1.492, 1.591) | 0.844 (0.791, 0.901) |
| March | 1.754 (1.638,1.879) | 1.608 (1.551, 1.668) | 1.091 (1.009, 1.179) | 1.411 (1.322,1.505) | 1.725 (1.662, 1.790) | 0.818 (0.759, 0.881) |
| April | 1.935 (1.790,2.091) | 1.765 (1.693, 1.839) | 1.096 (1.004, 1.197) | 1.517 (1.411,1.632) | 1.909 (1.831, 1.992) | 0.795 (0.731, 0.864) |
| May | 2.112 (1.939,2.302) | 1.918 (1.832, 2.008) | 1.101 (1.000, 1.214) | 1.620 (1.495,1.756) | 2.092 (1.996, 2.192) | 0.775 (0.706, 0.850) |
| June | 2.290 (2.085,2.514) | 2.070 (1.970, 2.175) | 1.106 (0.995, 1.229) | 1.720 (1.577,1.877) | 2.274 (2.161, 2.393) | 0.757 (0.684, 0.837) |
